# Self-perceived memory loss is associated with an increased risk of hip fracture in the elderly: a population-based NOREPOS cohort study

**DOI:** 10.1186/s12877-015-0135-8

**Published:** 2015-10-23

**Authors:** M. Garcia Lopez, T. K. Omsland, A. J. Søgaard, H. E. Meyer

**Affiliations:** Department of Community Medicine, Institute of Health and Society, University of Oslo, PO Box 1130, N-0318 Oslo, Norway; Department of Endocrinology, Morbid Obesity and Preventive Medicine, Oslo University Hospital, Oslo, Norway; Division of Epidemiology, Norwegian Institute of Public Health, Oslo, Norway

**Keywords:** Memory loss, Hip fracture, Elderly, Cohort study, Population-based

## Abstract

**Background:**

An early detection of memory loss may hold great value as a predictor for dementia. Dementia has already been associated with higher risk of hip fracture. Our aim was to examine the prospective association between self-reported memory-loss and the risk of subsequent hip fracture in the elderly.

**Methods:**

A population-based prospective cohort study design was used. Information on four self-perceived memory loss questions was obtained from questionnaires in 3 health surveys performed in Norway during the years 2000–2001. A total of 7154 men and 2462 women aged 67–77 years old were followed for a median of 7.8 years. Hip fracture information (*n* = 287 in men, and *n* = 237 in women) was obtained from NORHip (a database including all hip fractures treated in Norway from year 1994). Cox survival analysis was performed to estimate HR (hazard ratio).

**Results:**

The risk of sustaining a hip fracture were higher in those who reported to forget things they had just heard or read, with a HR of 1.52 (1.19--1.95) in men and HR 1.60 (1.23--2.07) in women after adjustment for relevant confounders. Women reporting to forget where they had put things also had higher risk of later hip fracture with a HR of 1.58 (1.20--2.07). Answering yes in both questions showed stronger association with sustaining a first hip fracture compared with those who gave a negative response in both questions, with a multivariate adjusted HR of 1.41 (IC 95 % 1.06--1.88) in men and 1.90 (IC 95 % 1.39--2.60) in women. The two last questions did not show a significant association with hip fracture.

**Conclusions:**

There was a higher risk of hip fracture in elderly who reported self-perceived memory loss. Due to the serious implications of sustaining a hip fracture, early detection of risk groups is important for preventive interventions.

## Background

Hip fracture is a well-known public health problem with high incidence in Western societies [1]. In addition to the clinical and social consequences [2], there is a substantial increased mortality following a hip fracture [3, 4]. With an expected future growth of the older population [5], conditions suffered by the elderly are gaining importance. Among them, dementia is one of the most relevant and due to the lack of treatment; more studies are focused on the pre-dementia stages in an effort to prevent the natural evolution or at least to slow it down. Mild cognitive impairment (MCI) and subjective cognitive impairment (SCI) are measurable factors included in these stages. MCI is a syndrome affecting cognitive functions, but without reaching diagnosis criteria for dementia [6]. SCI is a condition based on answers to questions about memory complaints. However, a standardized criteria is still needed to define SCI [7]. Both conditions have been described as predictors for dementia [8, 9]. Previous studies have shown that patients suffering from dementia or MCI have higher risk of experiencing a hip fracture and falling [10, 11]. Several studies have showed association between cognitive disorders and hip fractures in the post-surgical time [12, 13]. In this study, we investigate the prospective association between self-perceived memory loss and the risk of sustaining a first hip fracture during follow up time by using four question related to memory included in a multipurpose health screening. To the best of our knowledge, no other studies have addressed this association before.

## Methods

### Subjects (participants)

We included women and men aged 67–77 years participating in regional health surveys in Oslo (Oslo II and HUBRO) and in two counties north of Oslo (OPPHED) which were carried out during the years 2000–2001 [14]. The Norwegian Institute of Public Health conducted all the screenings following a similar protocol with standardized anthropometric measurements and questionnaire data. One of the studies, HUBRO, is described in more detail before [14, 15]. All studies are part of Cohort of Norway (CONOR) [16]. The studies described in this paper, included participants in several age ranges at time of examination: OPPHED (75–76, 60–61, 45–46, 40–41, 30–31, 15–16 years), Oslo II (67–77 years), and HUBRO (75–77, 59–61, 45–46, 40–41, 30–31, 15–16). In the present study we included persons aged 67–77 years at examination due to the lower incidence of hip fracture and cognitive impairment in the younger age groups. In total, 10449 individuals matched the requirements. Of these, 833 had missing information regarding the exposure variables, leaving a total of 9616 subjects in the main analyses (74 % of which were men).

### Assessment of memory

The three surveys were designed as multipurpose health studies including information about comorbidities, lifestyle and socio-economic factors.

A medical examination with anthropometric measurements was also carried out by trained personal. Four memory-related questions based on The Canberra community version of the Geriatric Mental State Examination (GMS, 10) were posed to the participants in the surveys. An affirmative answer to one or more of these questions describes the personal perception of memory loss. However, this does not mean that an objective cognitive disorder is present, as no measurements with diagnosis tests were performed in the subjects.

### Hip fracture assessment

Data on first hip fracture occurring form baseline and during follow-up was obtained by linkage to the NORHip (a NOREPOS database) [17], containing data on all hip fractures treated at Norwegian hospitals from 1994 to 2008. NOREPOS (The NORwegian EPidemiologic Osteoporosis Studies) is a research collaboration between four Norwegian epidemiologic osteoporosis studies, based on large population surveys in four districts of Norway (Tromsø, Nord-Trøndelag, Hordaland, Oslo). Diagnosis codes of hip fracture (International Classification of Diseases, Tenth Revision (ICD-10) codes: S72.0-S72.2, surgery procedures codes and time between hospitalizations was taken into account to avoid recording re-hospitalizations as new hip fractures [3].

### Potential confounders (covariates)

The following covariates, thought to be relevant to our study, were included: *age* (years), *gender*, *smoking* (daily smoking vs. the rest), *physical activity* (a combination of two questions: light and hard physical activity, with a result of 8 categories) used dichotomized with score 1–2 as one category (nothing or very light activity) versus the rest, *self-perceived health* (by answering the question: how is your current health? with four possible responses: poor, not very good, good and very good) dichotomized into poor-not very good vs. the rest. BMI (body mass index) was calculated from height and weight measures, using the formula weight (kg) divided by the square of height (m^2^). BMI was used as a continuous and as a categorical variable (divided in four categories: BMI < 22 kg/m^2^, 22–24 kg/m^2^, 25–29 kg/m^2^, ≥30 kg/m^2^). The participants also responded to questions concerning: (1) use of *blood pressure medication, lipid lowering drugs, antidepressants, anxiolytics* and *sleeping pills*. These variables were dichotomized into: daily intake vs. other categories, (2) chronic conditions: *stroke, myocardial infarction and diabetes*, which were dichotomized into yes (if a history of them was reported by the patient) or no, (3) *Educational level* (based on years of education), and (4) *self-reported alcohol intake* reported in 6 categories based on frequency of consumption (several times per week, once per week, 2–3 times per month, once or less per month, abstainer, never).

### Statistical analysis

Baseline differences between those who sustained a hip fracture during follow-up, and the rest were tested with the Chi–squared test for categorical variables, and *t* test for continuous variables. Observation time was calculated from the health screening to fracture or censoring (death, emigration or end of study). Data on death and emigration was obtained by linkage to the National Population Register. Cox proportional hazards regression was used to estimate the association between self-perceived memory loss and having a first hip fracture as hazard ratios (HR) with confidence interval (CI). Schoenfeld Residuals plots were used to check proportionality assumption, and no violation of proportionality was detected. In the regression models, we first adjusted for age and subsequently included self-perceived health and use of psychotropic medication (antidepressants, anxiolytics and sleeping pills). Final adjustments were performed including the rest of the variables considered as potential confounders. We performed all analyses twice including missing information as separate categories in the covariates and also by imputing missing information with multiple imputation (data not shown), showing no substantial difference in results. STATA statistical software, version 14.0 (Stata Corp.Texas.USA) was used in the analysis.

### Ethical considerations

The study and data linkage were approved by the Regional Committee for Medical and Health Research Ethics (South- East) and the Norwegian Data Protection Authority, and meets the standards of the Declaration of Helsinki (2014)[18] . All participants gave their written informed consent.

## Results

In the 7154 men and 2462 women included, median time of follow-up was 7.8 years and mean age at examination was 72.6 years in men and 75.9 years in women. During follow-up time, 287 men and 237 women sustained a hip fracture.

As can be seen in Table 1, women and men who sustained a hip fracture reported poorer self-perceived health status, had a lower BMI and used more antidepressants, anxiolytics and sleeping pills compared to the individuals not sustaining a hip fracture. They also reported chronic conditions in higher proportions, and in men, hip fracture patients were older, smoked more and were less physical active. In Cox analyses (Table 2), participants answering yes to question 1 (“Do you forget things you have just heard or read?”) had higher risk of sustaining a hip fracture with a HR = 1.52, (95 % CI 1.19--1.95) in men and HR = 1.60 (95 % CI 1.23--2.07) in women, after adjustment for all covariates described in *covariates* section above. There was no significant association in men who reported to forget where they had put things and risk of hip fracture (Q2), while women who answered yes to the this question showed higher risk of sustaining a hip fracture (HR = 1.58, 95 % CI 1.20--2.07) in the final model including all covariates. However, there was no significant association between hip fracture and question 3, ” Is it more difficult to remember things now compared with before?” (HR: 1.12, IC 95 %: 0.85--1.49 in men, HR: 1.24, IC 95 % 0.89--1.73 in women) and question 4, “Do you more often write a memo now than before?” (HR: 1.15 (IC 95 %: 0.91--1.47) in men, HR: 1.01 IC 95 % 0.78--1.30) in women).

**Table 1:** Baseline characteristics of the study population according to hip fracture in men and women (*n*=9.616). A NOREPOS study.

	Men		Women	
	With hip fracture	Without hip fracture	With hip fracture	Without hip fracture
Participants, number (%)	287 (4.0 %)	6.867 (96.0 %)	237 (9.6 %)	2225 (90.4 %)
Age, years, mean (±SD)	73.7** (±2.9)	72.5 (±3.1)	76.0 (±0.6)	75.9 (±0.5)
BMI, mean (±SD)	25.3** (±3.5)	26.4 (±3.3)	25.2** (±4.0)	26.5 (±4.3)
Education level, years, mean (±SD)	11.8 (±3.8)	11.9 (±3.9)	10.1 (±3.0)	10.2 (±3.2)
Self‐perceived health (poor--not very good) (% yes)	39.4**	28.0	51.7*	43.6
Smoke daily (% yes)	27.6**	18.5	19.0	14.8
No/little physical activity (% yes)	9.7*	6.5	11.8	13.3
Alcohol consumption Several times per week (%)	30.6	32.9	14.7	14.6
Medication intake (% answering yes)				
Blood pressure medication, daily	35.4	34.0	33.2	37.3
Lipid lowering drugs, daily	19.7	19.4	22.0	19.1
Sleeping pills, daily/every week	14.9**	8.1	32.1**	21.9
Anxiolytics daily/every week	9.2**	4.4	17.7**	11.6
Antidepressants daily/every week	4.9*	2.8	14.6**	7.6
Chronic conditions (% answering yes)				
Stroke, (%)	13.6**	7.6	8.1	7.6
Myocardial Infarction (%)	13.6	12.9	9.2*	6.1
Diabetes (%)	9.8	7.4	7.4	6.4
Questions related to memory (% answering yes)				
* Do you forget things you have just heard or read? (Q1)*	36.3**	24.6	41.7**	28.6
* Do you forget where you have put things? (Q2)*	48.2	43.6	66.1**	52.5
* Is it more difficult to remember things now compared with before? (Q3)*	80.6	75.8	83.1	80.0
* Do you more often write a memo now than before? (Q4)*	37.3	33.1	46.2	46.7

BMI: body mass index

Differences were tested by using a chi--square test for categorical variables, and t test for continuous variables. *p‐value <0.05 **p‐value <0.01

**Table 2:** Hazard ratios (HR) with 95 % confidence interval (CI) of hip fracture in men and women aged 67--77 years according to self‐perceived memory loss and adjusted for covariates. A NOREPOS study.

Self‐perceived memory loss	Number (*n*)	Hip fracture *n* (%)	HR (CI 95 %) Model (a)	HR (CI 95 %) Model (b)	HR (CI 95 %) Model (c)	HR (CI 95 %) Model (d)
Question 1: Do you forget things you have just heard or read?						
Men						
No	5369	184 (3.4 %)	1.00 (ref)	1.00 (ref)	1.00 (ref)	1.00 (ref)
Yes	1785	103 (5.8 %)	1.68 (1.32--2.14)	1.57 (1.23--2.00)	1.56 (1.22--2.00)	1.52 (1.19--1.95)
Women						
No	1727	137 (7.9 %)	1.00 (ref)	1.00 (ref)	1.00 (ref)	1.00 (ref)
Yes	737	100 (13.6 %)	1.77 (1.37--2.29)	1.68 (1.30--2.18)	1.62 (1.25--2.11)	1.60 (1.23--2.07)
Question 2: Do you forget where you have put things?						
Men						
No	4066	153 (3.8 %)	1.00 (ref)	1.00 (ref)	1.00 (ref)	1.00 (ref)
Yes	3088	134 (4.3 %)	1.12 (0.89--1.41)	1.07 (0.84--1.35)	1.06 (0.84--1.34)	1.06 (0.84--1.34)
Women						
No	1174	83 (7.1 %)	1.00 (ref)	1.00 (ref)	1.00 (ref)	1.00 (ref)
Yes	1288	154 (12.0 %)	1.75 (1.34--2.29)	1.67 (1.27--2.18)	1.61 (1.23--2.11)	1.58 (1.20--2.07)
Question 1+2						
Men						
No to both questions	3727	131 (3.5 %)	1.00 (ref)	1.00 (ref)	1.00 (ref)	1.00 (ref)
Yes to both questions	1446	81 (5.6 %)	1.56 (1.19--2.06)	1.44 (1.09--1.91)	1.43 (1.08--1.90)	1.41 (1.06--1.88)
Women						
No to both questions	1073	75 (7.0 %)	1.00 (ref)	1.00 (ref)	1.00 (ref)	1.00 (ref)
Yes to both questions	636	92 (14.5 %)	2.17 (1.60--2.94)	2.04 (1.50--2.76)	1.95 (1.43--2.66)	1.90 (1.39--2.60)

(a) Adjusted for age

(b) Adjusted for (a) and self--‐perceived health

(c) Adjusted for (b) and, use of antidepressants, use of anxiolytics and use of sleeping pills

(d) Adjusted for (c) and BMI, smoking, physical activity, stroke, myocardial infarction, diabetes , blood pressure medication, lipid lowering drugs, years of education and alcohol consumption

Based on these results, we decided to perform an analysis collapsing answers to question 1 and 2 into a new variable (Table 2). The risk of hip fracture was more than 50 % higher in men and more than doubled in women who answered yes to both questions compared to their counterpart’s answering no. This association was attenuated when adjusted for confounders, but was still associated with a statistically significant increased fracture risk of 41 % (95 % CI 6 %--88 %) in men and a 90 % (95 % CI 39 %--160 %) in women. As shown in Table 2, self-perceived health and psychotropic medication influenced on the estimated associations between the memory loss questions and hip fracture, whereas additional adjustment for blood pressure medication and lipid lowering drugs, stroke, diabetes, myocardial infarction, years of education and self-reported alcohol intake did not affect the estimates substantially.

## Discussion

The results of this study show a significantly higher risk of sustaining a hip fracture among individuals reporting self-perceived memory loss compared to those not reporting this problem (Table 2). Two of the four questions on memory function predicted hip fracture. There was no association between question 3 (Is it more difficult to remember things now compared with before?) and hip fracture. However, this question discriminated poorly as around 80 of all participants responded positive. Neither was there any significant association between question 4 and hip fracture, but it could be argued that writing a memo more often than before is not a direct question on self-perceived memory loss.

For question 2, we only found a significant association between self-perceived memory-loss and hip fracture in women and not in men for reasons which are not clear for us. However, the difference between the proportion of men answering yes to this questions and the men answering no, is not as large as in women. One potential explanation for this migh be a gender difference of symptom reporting, based on symptom perception [19].Analyses combining both questions rely upon the individual results in each of the questions, so it is expected to find a higher risk of sustaining a hip fracture in women reporting memory-loss than in men.

To the best of our knowledge this is the first study reporting an association between self-perceived memory loss and hip fracture in the elderly. However we have identified some studies, which have reported associations between mild cognitive impairment, and subjective cognitive impairment with higher risk of falling and poor balance control [10, 20]. The concept of subjective cognitive dysfunction has recently gained attention as it could be consider a pre-clinical stage in the sequence of dementia [21]. It can be defined as a cognitive decline, preceding mild cognitive impairment. Though, it is important to note that not all the individuals suffering from subjective cognitive impairment will develop dementia. Individuals suffering from subjective cognitive impairment usually present memory complaints and have poorer performance in diagnostic memory test (as Mini Mental State examination) [21]. In our study, as no formal mental tests were performed, we used the term self-perceived memory loss though we are aware that we may have included persons with cognitive impairment in our group.

A possible explanation for the association between self-reported memory loss and hip fracture might be related to an increasing number of falls. It has already been shown that dementia and mild cognitive impairment are two disorders associated with higher risk of falling, as they affect motor function and balancing [10]. Subjective cognitive impairment has also been related to spatial navigation and balance [20]. In this regard, self-perceived memory loss could be considered a sign of early cognitive dysfunction slightly affecting other neurological functions apart from memory. Medication intake is another fall-related explanation. Due to the age range of our study group, most individuals suffered from one or several chronic conditions. This usually implies taking prescribed drugs that might increase the risk of falling. Psychotropic drugs have presented strong association with hip fracture in previous research [22]. In our study, adjustment for the use of antidepressants, anxiolytics and sleeping pills slightly decreased the risk of fracture in both genders, but the estimates were still statistically significant. Blood pressure medication and lipid lowering drugs have been related to falls, but usually presenting weak or null association with hip fracture [23]. In our study, taking these two groups of medication did not influence on our results.

Adjustment for self-perceived health attenuated the association between self-perceived memory loss and hip fracture to some extent. This indicates that a high percentage of the individuals reporting self-perceived memory loss also considered that they had poor or not very good health.

One of the main strengths is having a large study population followed with respect to hip fracture by data linkage to a nationwide validated hip fracture database (NORHip). The incidence of hip fracture in men is lower than in women [1]. However, due to a larger number of men in our study, we had the chance to study the association with similar statistical power in both genders. Another strength is using as exposure variables two memory-related questions, which are included as items in a validated questionnaire toll for the screening of cognitive functions. One limitation of this study is the inclusion of mainly white individuals with a limited age range (67–77). We can thus not generalize the results to individuals with other age or ethnic background. Further research lines imply wider studies exploring the association between memory loss and hip fracture, including mediating factors. This could for example include Vitamin B12, folate and homocysteine blood levels as they have been related both to dementia [24], and hip fracture [25, 26]. Development of validated tools to characterize more precisely the existence of early cognitive dysfunction is also warranted as this can imply higher risk of suffering hip fracture.

## Conclusion

In this large study among elderly Norwegians participating in population based health screenings, we found that questions indicating self-perceived memory loss predicted future hip fracture, also after adjustment for relevant confounders.

## Abbreviations

BMI: Body mass index
CI: Confidence interval
HR: Hazard ratio
MCI: Mild cognitive impairment
NOREPOS: The NORwegian EPidemiologic Osteoporosis Studies
NORHip: The NOREPOS Hip Fracture Database
SCI: Subjective cognitive impairment
